# Supplementary figures and images for: APC Mutation Marks an Aggressive Subtype of BRAF Mutant Colorectal Cancers
Source: Cancers (Basel). 2020 May 6;12(5):1171. doi: 10.3390/cancers12051171 (PMC7281581; doi:10.3390/cancers12051171)

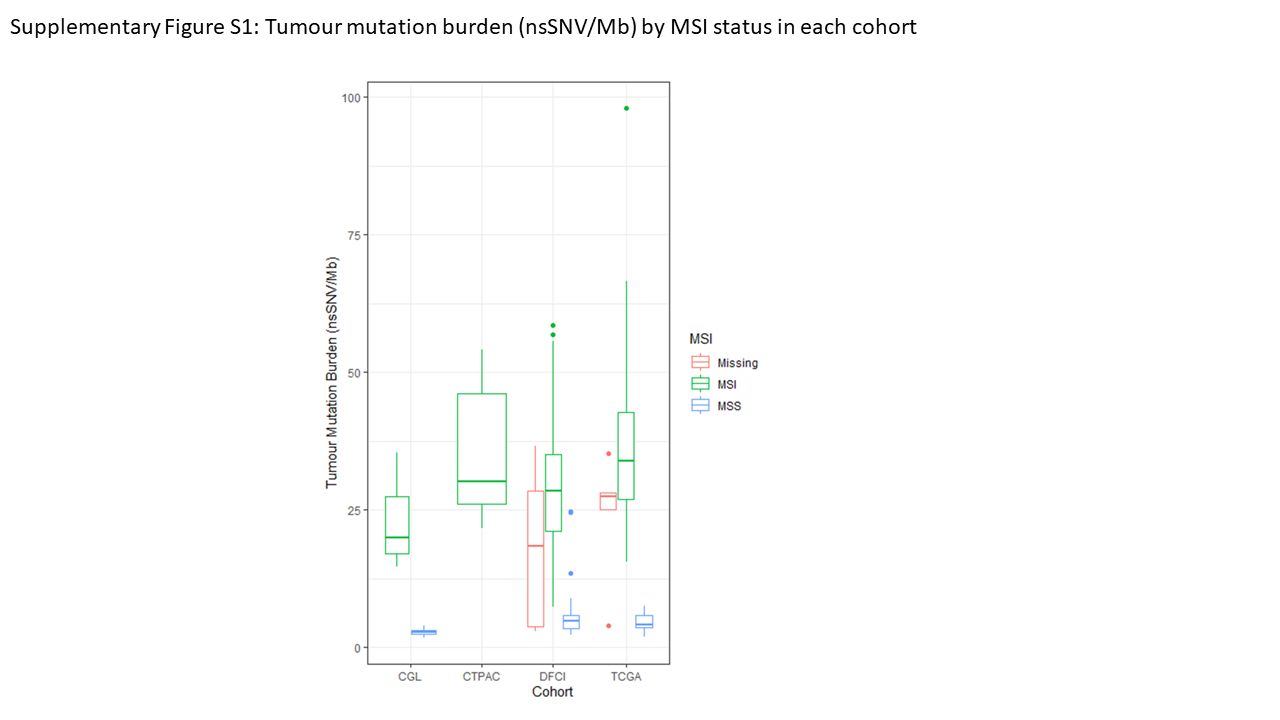

Supplement: Supplementary file 1 [file cancers-12-01171-s001.zip › Supplementary Figure S1.png]

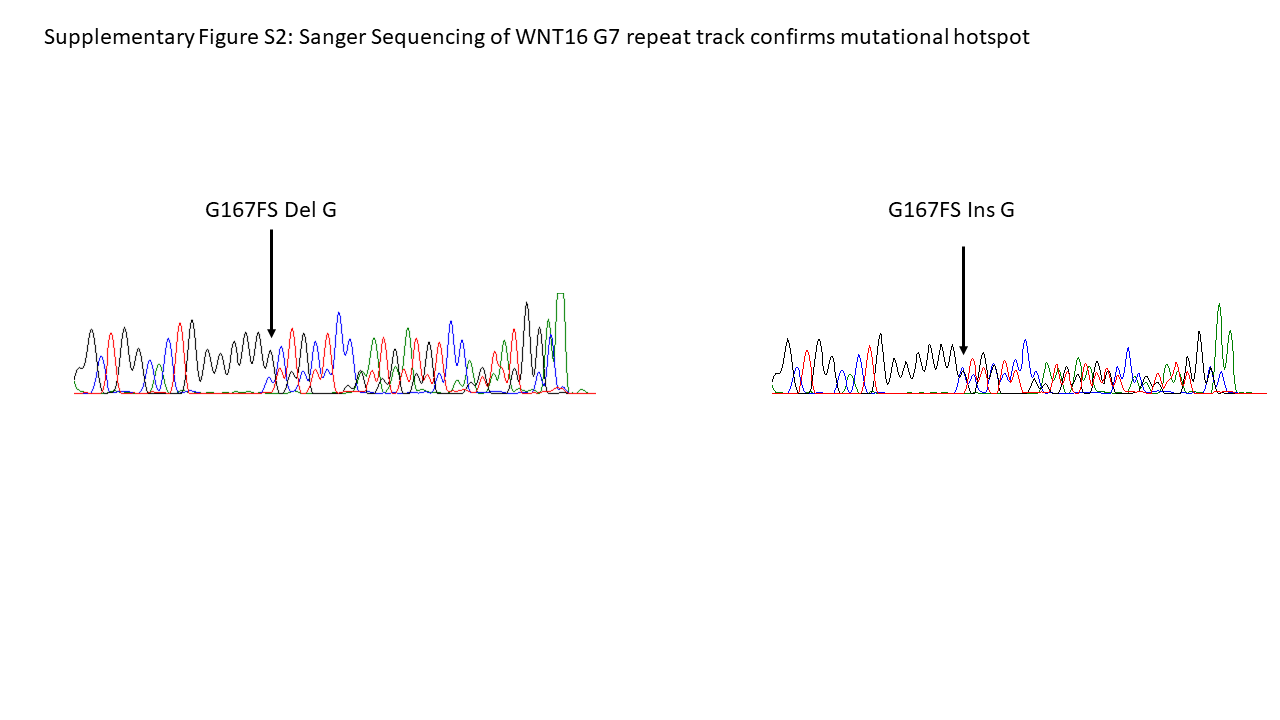

Supplement: Supplementary file 1 [file cancers-12-01171-s001.zip › Supplementary Figure S2.png]

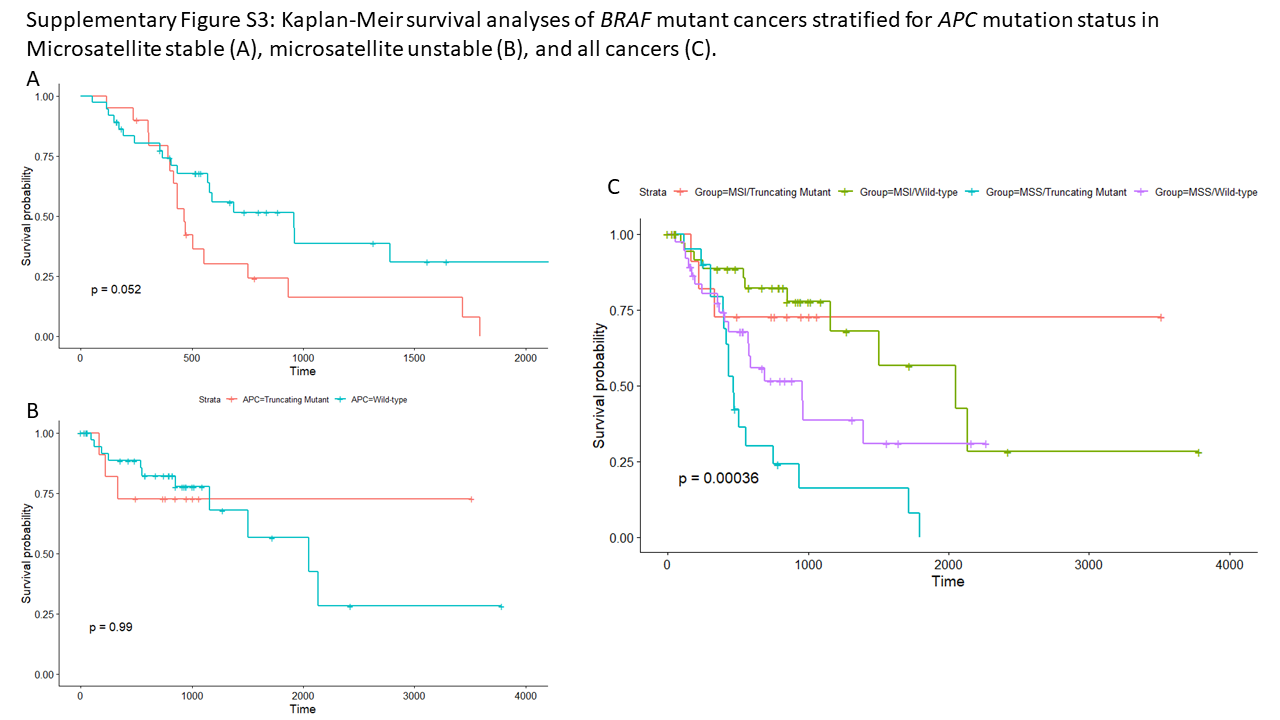

Supplement: Supplementary file 1 [file cancers-12-01171-s001.zip › Supplementary Figure S3.tif]
